# Domestic

**Published:** 1841-10-30

**Authors:** 


### DOMESTIC.

*Amputation of the Thigh.*—We extract with pleasure, from our worthy contemporary of the Boston Journal, the following letter from Dr. Trowbridge. The statistics of amputation in this country derive additional interest at the present moment from the light recently thrown upon the question of the relative mortality of operations in France and the United States, by the labours of Dr. Norris, of this city, and others. A body of well authenticated surgical statistics of this character, drawn from various sections of the country, in aid of the observations made at the various hospitals, would probably lead to results important to the improvement of hospital discipline as well as private practice.

Several valuable facts are noted in the letter, throwing light on the chances of life after amputation in fungus hæmatodes, white swelling, syphilis and scrofula. We plead guilty to some surprise in observing that twenty-six of the eighty-five cases of amputation were performed on account of necrosis; and that so many as twenty-three patients were affected with necrosis involving the knee joint should occur in the private practice of a single individual. Is this owing to an unusual prevalence of diseases of the bones in the particular neighbourhood of the writer? With us true necrosis, actually involving joints, is very rare, and amputation for this disease uncommon. We ask this question, not censoriously, but in good faith, as the terminology and diagnosis of diseases of the bones is still in much confusion.

Four cases are classed under the head of incised wounds dividing arteries and nerves, and three of the operations were performed for aneurisms of the arteries of the thigh. Such instances—particularly the former—could not be devoid of further interest if stated in detail, and we hope that Dr. Trowbridge will favour the profession, through the same or some other convenient channel, with a more complete history of those cases, if sufficient notes of them are still in existence.

Sir,—Within a few days past I have made my eighty-fourth and fifth operations of amputation of the thigh. All these operations have been made in my private practice, during the period of 34 years. The cases occurred principally in the northern portion of the State of New York. I made many amputations of the thigh, while attached to the U. S. Army, during the late war. I cannot state the number, or detail their causes. I am able to state the causes which made amputation necessary in my private practice, and the results following the operations. Eleven of these operations were on females, and seventy-four on males.

| | | |
|---|---|---|
| 2 | Cases of | Fungus hæmatodes. |
| 3 | " | Necrosis, under the age of 10 years |
| 2 | " | Osteo-sarcoma. |
| 3 | " | White swelling of the knee-joint. |
| 1 | " | Periostosis and destruction of the tissues of the knee-joint. |
| — | | |
| 11 Females. | | |
| 23 | Cases of | Necrosis, commencing on the femur, or tibia, and involving the knee-joint. |
| 7 | " | White swelling of the knee-joint. |
| 6 | " | Spina ventosa of the tibia, involving the knee-joint. |
| 10 | " | Fungus hæmatodes, principally located in the lower leg. |
| 8 | " | Compound fractures of the thigh, with contusions of the knee and leg. |
| 4 | " | Divisions of arteries and nerves by incised wounds. |
| 3 | " | Aneurisms of arteries of the thigh |
| 8 | " | Mortification, following contusions of the thigh and leg. |
| 5 | " | Fractures of the tibia, with dislocation of the ankle-joint, and contusion of the tissues, producing tetanus and mortification. |
| — | | |
| 74 Males. | | |

There was an entire recovery in all the female cases, from the operation of amputation. Those with fungus hæmatodes died within three years after amputation with tuberculated state of the lungs.

Out of the 74 cases of amputation of the thigh on the males, two died within twenty-four hours after the operation—one from secondary hæmorrhage, and the other from extreme feebleness at the time of the operation; no reaction followed. Nine died from the suppurative process on the stump following the operation, occasioned by venereal and scrofulous taints of the system; remained in a feeble state from five to twenty months, and died. The other 54 entirely recovered from the operation, and were healthy.

Those with fungus hæmatodes, who suffered amputation for that affection, passed under visceral disease, (the lungs and mesenteric glands) and died within four years after the operation, though apparently healthy for the first year or two.

Four that suffered with white swellings, after amputation declined, and perished with pulmonary consumption after four years.

For the first few years, in operating, I was governed by the directions laid down by C. Bell in his first work on operative surgery. In all cases where a limb was to be lost, from any sudden accident, I advised and practised immediate amputation, or before much reaction or inflammation commenced. I made the operation by a circular incision, as directed by Mr. Bell, turned back the integuments, and divided the muscles by applying the knife under the supported integuments; commencing on the inner margin of the vastus internus, and carrying the knife obliquely upward, making one sweep through the whole muscles to the bone, which bared the bone two or three inches higher than by the perpendicular incision. These incisions were expedited by an assistant elevating the parts as fast as they were divided, and using a retractor for the application of the saw. I usually dissected the muscles from the bone about an inch, and removed the periosteum only at a point for the saw. This mode of operating generally occupied from three to five minutes, and the dissection and turning up of the integuments gave the patient great pain. To secure the arteries, I always used the tenaculum of a small size, and a ligature of two flaxen threads well waxed, and left flat, on the large arteries, and one thread on the small ones, and secured every artery that threw out blood. After this, brought down the integuments and muscles over the bone, and secured with adhesive straps, six inches long; placed thick dossils of lint over these, and compresses and roller over the whole, so as to make an easy support to the parts. If spasms followed upon the stump, producing much agony, I let blood and gave opium. I used the animal ligature in some cases, tortion, and other means recommended for securing arteries, but have always succeeded best with the thread.

Although I always had success with the circular incision, and process, as mentioned in the first thirty cases of amputation, yet I afterwards occasionally operated by making two flaps, by dividing the integuments in a circular manner with a large scalpel, down to the muscles, and turning them back, and then dividing the muscles as in the circular operation. This mode subjected me to a serious difficulty.—The bone would incline to rise and protrude towards the opening at the upper part of the limb.

In a majority of the last twenty cases of amputation of the thigh, I have operated by making the flap-amputation; passing a narrow double-edged knife, and cutting from the bone outward on each side, making two flaps. These are semi-circular, their convexities extending in a parallel manner forward, and their terminations meeting at the upper and lower surface of the limb, where the knife entered and passed out.

I have operated a few times by cutting in a semicircular manner from the surface to the bone, making two flaps. This gives more pain to the patient than the mode of cutting from the bone outward. The painful part of the operation, viz., cutting the soft parts, can be made in the first manner very quick, and the patient saved from much distress. The time occupied by me generally, in ordinary cases, is from thirty to forty seconds, and in every instance a quick union of the flaps and sound state followed by the first intention.

I have met with no instance of nervous irritation, or difficulty in securing arteries, after operating in this manner, as recently reported by some. In emaciated cases, I should still prefer the circular operation; but in this matter I would advise every operator to consider the nature of his case, and adjust means accordingly.

AMASA TROWBRIDGE.

*Watertown, N. Y., Oct.*, 1841.

---

INTERMENTS *in the City and Liberties of Philadelphia, from the 16th of October to the 23d.*

| Diseases. | Adults. | Children. | Diseases. | Adults. | Children. |
|---|---|---|---|---|---|
| Apoplexy | 1 | 1 | *Brought forward*, | 39 | 27 |
| Burns, | 0 | 1 | Intemperance, | 3 | 0 |
| Cancer, | 1 | 0 | Marasmus, | 2 | 3 |
| Croup, | 0 | 2 | Morbus Brightu, | 1 | 0 |
| Congestion of brain, | 0 | 1 | Old age, | 4 | 0 |
| Childbed, | 1 | 0 | Palsy, | 1 | 0 |
| Consumption of the lungs, | 14 | 2 | Pleurisy, | 1 | 0 |
| Convulsions, | 1 | 4 | Rupture of Urethra, | 1 | 0 |
| Diarrhœa, | 2 | 0 | Small pox, | 4 | 5 |
| Dropsy, | 0 | 1 | Still-born, | 0 | 7 |
| —— head, | 0 | 1 | Suicide, | 1 | 0 |
| Disease of the Brain, | 1 | 1 | Tetanus, | 1 | 0 |
| —— Heart, | 1 | 0 | Tabes Mesenterica, | 0 | 1 |
| —— Bowels, | 1 | 0 | Total, 101— | 58 | 43 |
| Drowned, | 2 | 0 | | | |
| Dysentery, | 0 | 2 | | | |
| Debility | 0 | 1 | | | |
| Epilepsy, | 1 | 0 | | | |
| Fever, Remittent, | 1 | 0 | | | |
| —— Bilious, | 2 | 0 | | | |
| —— Typhus, | 1 | 1 | | | |
| —— Typhoid, | 1 | 0 | | | |
| —— Scarlet, | 0 | 4 | | | |
| Fungus, | 0 | 1 | | | |
| Inflammation, | 1 | 0 | | | |
| —— Breast, | 0 | 1 | | | |
| —— Lungs, | 3 | 1 | | | |
| —— Stomach, | 1 | 0 | | | |
| —— Bowels, | 2 | 1 | | | |
| —— Liver, | 1 | 0 | | | |
| —— Uterus | 1 | 0 | | | |
| *Carried forward*, | 39 | 27 | | | |

Of the above, there

| | |
|---|---|
| were under 1 year, | 22 |
| From 1 to 2 | 6 |
| 2 to 5 | 10 |
| 5 to 10 | 2 |
| 10 to 15 | 0 |
| 15 to 20 | 3 |
| 20 to 30 | 13 |
| 30 to 40 | 13 |
| 40 to 50 | 13 |
| 50 to 60 | 4 |
| 60 to 70 | 5 |
| 70 to 80 | 8 |
| 80 to 90 | 2 |
| Total, | 101 |

